# Angiogenic Potential of Bone Marrow Derived CD133^+^ and CD271^+^ Intramyocardial Stem Cell Trans- Plantation Post MI

**DOI:** 10.3390/cells9010078

**Published:** 2019-12-27

**Authors:** Sarah Sasse, Anna Skorska, Cornelia Aquilina Lux, Gustav Steinhoff, Robert David, Ralf Gaebel

**Affiliations:** 1 Department of Cardiac Surgery, Rostock University Medical Center, 18059 Rostock, Germany; sarah-sasse@t-online.de (S.S.); anna.skorska@med.uni-rostock.de (A.S.); cornelia.lux@gmx.de (C.A.L.); gustav.steinhoff@med.uni-rostock.de (G.S.); ralf.gaebel@med.uni-rostock.de (R.G.); 2 Department Life, Light & Matter (LL&M), University of Rostock, A.-Einstein-Str. 25, 18057 Rostock, Germany

**Keywords:** bone marrow stem cells, angiogenesis, myocardial infarction, cardiac regeneration

## Abstract

Background: Bone marrow (BM)-derived stem cells with their various functions and characteristics have become a well-recognized source for the cell-based therapies. However, knowledge on their therapeutic potential and the shortage for a cross-link between distinct BM-derived stem cells, primed after the onset of myocardial infarction (MI), seems to be still rudimentary. Therefore, the post-examination of the therapeutic characteristics of such primed hematopoietic CD133^+^ and mesenchymal CD271^+^ stem cells was the object of the present study. Methods and Results: The effects of respective CD133^+^ and CD271^+^ mononuclear cells alone as well as in the co-culture model have been explored with focus on their angiogenic potential. The phenotypic analysis revealed a small percentage of isolated cells expressing both surface markers. Moreover, target stem cells isolated with our standardized immunomagnetic isolation procedure did not show any negative alterations following BM storage in regard to cell numbers and/or quality. In vitro network formation relied predominantly on CD271^+^ stem cells when compared with single CD133^+^ culture. Interestingly, CD133^+^ cells contributed in the tube formation, only if they were cultivated in combination with CD271^+^ cells. Additional to the in vitro examination, therapeutic effects of the primed stem cells were investigated 48 h post MI in a murine model. Hence, we have found a lower expression of transforming growth factor βeta 3 (TGFβ3) as well as an increase of the proangiogenic factors after CD133^+^ cell treatment in contrast to CD271^+^ cell treatment. On the other hand, the CD271^+^ cell therapy led to a lower expression of the inflammatory cytokines. Conclusion: The interactions between CD271^+^ and CD133^+^ subpopulations the extent to which the combination may enhance cardiac regeneration has still not been investigated so far. We expect that the multiple characteristics and various regenerative effects of CD271^+^ cells alone as well as in combination with CD133^+^ will result in an improved therapeutic impact on ischemic heart disease.

## 1. Introduction

In order to cover up the deficit of regenerative potential, new treatment approaches for cardiac diseases are needed. Several studies suggest that approaches based on the application of stem cells may be the future strategy in order to compensate the regenerative deficiency of the heart. As myocardial infarction (MI) occurs due to the absent blood flow in the coronary arteries, the formation of new blood vessels would be an essential prerequisite for the regeneration. Yet, in the search for suitable stem cell subtypes, various other properties are of interest as well. This includes cells that interfere with pathological immune mechanisms as well as cellular mediators and chemokines that cause migration and homing processes. Besides, it is of the tremendous importance whether the cells are proofed to be safe in application. Furthermore, the cells can also be treated in advance in order to develop desirable properties. As the CD133^+^ stem cell therapies and other approaches with hematopoietic cells provide promising results, research has to examine more cell types in order to achieve additional effects and thus to further improve the therapeutic measures. Until now, no stem cell-related cardiac complications following intramyocardial transplantation of bone marrow (BM) derived stem cells have been reported so that the therapy is considered to be safe [1]. In addition, the BM also contains a population named after their ability to differentiate into various cell types of mesenchymal origin. These multipotent stem cells are expandable and modifiable in cell culture and offer several distinctive characteristics, which make them a promising therapeutic approach for damaged heart tissue [2]. Especially mesenchymal stem cells (MSC) are able to decrease infarct size and myocardial fibrosis [3]. Thereby, cardiac remodeling in total may be attenuated as well as the heart’s contractility improves [3]. Furthermore, MSC have paracrine effects that inhibit apoptosis of endothelial cells in hypoxic condition, increase their survival, and thus stimulate angiogenesis processes [4]. As another advantage, MSC do not express any antigens of histocompatibility and thus can be used in allogenic transplantation as well. Quite the contrary, these cells even have immunomodulatory paracrine effects that counteract inflammatory responses [5,6]. As inflammatory processes play a decisive role in the development of chronic heart diseases and have a negative influence on the heart function, these effects may also improve the therapy significantly. After the transplantation, MSC may be able to stimulate the production of reparative growth factors, inhibit local inflammatory response, and replace damaged cells [7]. Apart from these positive effects, difficulties with the practical application arise from the large heterogeneity of MSC as well. Thus, it is uncertain to predict which exact cell type arises from the MSC population after the application. Indeed, a study by Yoon et al. showed that the direct transplantation of unselected BM cells into the infarcted myocardium induces significant intramyocardial calcification [8]. This clearly demonstrates the research demands in order to guarantee the safety of MSC as therapeutic option. The development of methods for the identification and isolation of MSC subpopulations is crucial for a determined specific function and a successful clinical application. A currently much examined MSC subtype is characterized by the expression of the surface marker CD271. Several studies showed that this marker is one of the most specific concerning BM derived MSC [9]. Thus, there may be a particular suitability of CD271^+^ MSC for preselected cell products. CD271 enables prospective cell isolation of MSC from BM derived mononuclear cells (MNC) [10] and have been shown to be essential in the formation of the heart as they influence the survival of endothelial cells, vascular smooth muscle cells, and cardiac myocytes. Furthermore, these proteins regulate angiogenesis and vasculogenesis by autocrine and paracrine mechanisms [10,11,12].

Knowledge of the therapeutic potential attributable to distinct BM derived stem cell subtypes primed due to MI of the donors is still greatly lacking. Therefore, this study intends to examine the benefits of CD271^+^ and CD133^+^ stem cells. In this context, we have explored the effects of CD271^+^ cells alone as well as in combination with CD133^+^ with the main focus on their angiogenic potential (in vitro examination). Moreover, the therapeutic capacities of both primed BM stem cells were investigated following cardiac ischemia/reperfusion in mice.

## 2. Materials and Methods

### 2.1. Bone Marrow Aspiration

Informed donors gave written consent to the aspiration of their BM according to the Declaration of Helsinki. The ethical committee of the University of Rostock has approved the presented study (registered as no. A201023) as of 29 April, 2010. BM samples were obtained by sternal aspiration from patients undergoing coronary artery bypass grafting (CABG) surgery at Rostock University, Germany. Anticoagulation was achieved by heparinization with 250 i.E./mL sodium heparine (B. Braun Melsungen AG, Melsungen Germany).

### 2.2. Cell Isolation (Single and Dual Method)

Mononuclear cells (MNC) were isolated by density gradient centrifugation using Lymphocyte Separation Medium (LSM; 1.077 g/L, PAA Laboratories GmbH, Pasching, Austria). CD133^+^ and CD271^+^ stem cells were enriched by positive magnetic selection using the magnet activated cell sorting (MACS) system (Miltenyi Biotec, Bergisch Gladbach, Germany). In case of CD133^+^ direct labeling was applied and indirect labeling for CD271^+^ cells (CD271-APC/anti-APC-microbeads; all Miltenyi Biotec). Purified stem cells were appropriately employed in further in vitro and in vivo experiments. For the dual method, the CD133^+^ population was isolated from the CD271^–^ fraction and vice versa.

### 2.3. Phenotypical Characterization by Flow Cytometry

Cell fractions were suspended in cold MACS-buffer containing PBS, 2 mM EDTA and 0.5% BSA. To reduce unspecific binding, FcR blocking reagent (Miltenyi Biotec) was added to all samples. Cells were stained using the mouse anti-human-CD133-phycoerythrin (PE; 293C2), -CD34-fluorescein isothiocyanate (FITC) and -CD271-allophycocyanine (all Miltenyi Biotec), -CD45-allophycocyanin-H7 as well as -CD45-Horizon-V500 (both BD Biosciences, Heidelberg, Germany) following incubation for 10 min in the dark at 4 °C. For optimal multicolor setting and correction of the spectral overlap single stained mouse isotype antibodies were considered as controls. The gating strategy was performed with matched isotype/fluorescence minus one control. After performing antibody staining 15 µM 4′,6-Diamidino-2-phenylindole (DAPI, Thermo Fisher Scientific, Waltham, MA, USA.) was added, cells were incubated for 2 min and then immediately acquired by BD™ LSRII flow cytometer and data were analyzed using FACS-Diva software, version 6.1.2 (both Becton Dickinson, Franklin Lakes, NJ, USA). Purity and viability of all cell isolations were analyzed using near-IR live dead stain (Thermo Fisher).

### 2.4. Angiogenesis Assay

Freshly isolated stem cells were prepared at 4 °C in an extracellular growth matrix using 100 µL BD Matrigel^TM^ Matrix (BD Biosciences) and 100 µL complete endothelial growth medium (EGM-2, Lonza, Basel, Switzerland) per well of a 24-well-plate (Greiner Bio-One, Kremsmünster, Austria). For the assay 100,000 CD133^+^ or CD271^+^ cells (single model) or both cell types in a ratio of 50,000 cells each (co-culture model) were seeded per well. After 20 min polymerization of the matrix at 37 °C the matrix 200 µL EGM-2was added carefully onto each well. The medium was changed every two days for another 14 days.

### 2.5. Three Dimensional (3D) Microscopy Analysis

After two weeks the tube formation assays were conducted according to the criteria of network length and count of nodal points. For this, z-stack images were acquired using the Zeiss high-resolution microscope ELYRA PS.1 LSM 780 confocal imaging system and corresponding Zen2011 software (both Carl Zeiss AG, Oberkochen, Germany). Z-Stack images are transversal slice images (two-dimensional) of the 3D assay and thus allow representative analysis of their structures. For each well, five z-stacks with ten images were taken and included into a final interpretation. The evaluation was performed using the ImageJ free software (NIH, Bethesda, MD, USA). For better illustration, the different levels of the z-stack were marked in different colors. Likewise, the amount of nodal points was counted, respectively. In total, 900 images (*n* = 6) were analyzed and measured toward network length and count of nodal points.

### 2.6. Cell Tracking within Matrigel Matrix

In order to further investigate the cell networks accomplished in Matrigel matrix, immunofluorescence staining was carried out on angiogenesis assay. For better discrimination and alterations within the matrix, freshly isolated CD133^+^ cells were stained with the lipophilic cell permeable dye CFDA-SE as well as CD271^+^ cells with the red fluorescent lipophilic tracer PKH26 (both Sigma-Aldrich, Saint Louis, MO, USA). Additionally, both cell types were stained for nuclei discrimination with Hoechst 33324 (Thermo Fisher). Acquisition and analyzes were performed using the Axiovert 40 CFL fluorescence microscope with Axio Cam MRm ZEN software (both Carl Zeiss AG).

### 2.7. Immunofluorescence Staining within 3D Matrix

Mouse anti-human-CD29 allophycocyanin as well as -CD73-phycoerythrin antibodies (both BD Biosciences) were diluted with EGM-2 in 1:10 ratio and incubated with the cells for 30 min. Afterwards, the assays were washed with EGM-2. For each marker an isotype control was applied in the same way in order to obtain a negative control. Additionally, both cell types were stained with Hoechst 33324. The analysis was performed by means of the Zeiss high-resolution microscope ELYRA PS.1 LSM 780 confocal imaging system and corresponding Zen2011 software Z-stack images were used for 3D reconstructions.

### 2.8. Gene Expression Analysis by Quantitative Real-Time-PCR

Cells derived from the single and co-culture models were collected after the termination of the angiogenesis assay and have undergone lysis in TRIzol^®^ reagent (Thermo Fisher). RNA was extracted following the manufacturer’s instructions. For reverse transcription of total RNA amount (2 µg) and cDNA synthesis, SuperScript^®^ III Reverse Transcriptase (Thermo Fisher) and oligo (dT)_15_ Primers (Promega, Fitchburg, WI, USA) were used. Quantitative real time-PCR was performed with StepOnePlus^TM^ Real-Time PCR System (Applied Biosystems, Waltham, MA, USA) in TaqMan^®^ Universal Master Mix (Thermo Fisher) according to the instructions of the manufacturer. The expression of the housekeeping gene ribosomal protein, large, P0 (human RPLP0, TaqMan^TM^ VIC^®^ Endogenous Control 4310879E) was used on each cell type. Similarly, human *ACTA* (alpha smooth muscle actin) (TaqMan^®^ Assay ID: Hs00909449_m1, FAM-MGB), *NGFR* (nerve growth factor receptor) (TaqMan^®^ Assay ID: Hs00609976_m1, FAM-MGB) and *vWF* (von Willebrand factor) (TaqMan^®^ Assay ID: Hs01109446_m1, FAM-MGB, all Thermo Fisher) were analyzed in duplicates and normalized to RPLP0. Negative controls were included in each assay. Cycle thresholds (C_T_) for single reactions were determined with StepOne™ Software 2.0 (formula: ΔC_T mean_ = C_T mean_ − C_T mean RPLP0_).

### 2.9. Animals

All animal procedures were in conformity with the guidelines from Directive 2010/63/EU of the European Parliament on the protection of animals used for scientific purposes. The federal animal care committee of LALLF Mecklenburg-Vorpommern (Germany) approved the study protocol (approval number LALLF M-V/TSD/7221.3-1.1-088/11). Severe Combined Immunodeficiency beige mice (SCID *beige*; strain CB17.Cg-*Prkdc^scid^Lyst^bg-J^*/Crl, female, 22 ± 2 g, Charles River, Sulzfeld, Germany) were randomly assigned to 4 groups: Healthy control (SHAM, *n* = 3), two MI groups with implanted human stem cells of the respective source (MI133, MI271 each *n* = 3) and untreated MI control group (MIC *n* = 3).

### 2.10. Generation of Reperfused MI in Mice and Stem Cell Implantation

Mice were anesthetized with 50mg/kg Pentobarbital (Vetmedica GmbH, Ingelheim, Germany) intraperitoneal injection. After thoracotomy and preparation, the left anterior descending coronary artery (LAD) was ligated. After 45 min each mouse received an intramyocardial cell injection. For cell treatment, 100,000 stem cells were suspended in 10 µL PBS containing 0.5% BSA and 2 mM EDTA and mixed with an equal amount of reduced growth-factor BD Matrigel^TM^ Matrix. The same experimental set-up was applied to control groups using cell suspension buffer and Matrigel^TM^ for application. Four injections, 5 µL each were given along the border of the blanched myocardium. Subsequently, the ligation was removed. Healthy control (SHAM)-operated mice underwent identical surgical procedures without left anterior descending coronary artery (LAD)-ligation.

### 2.11. Organ Harvesting

Mice underwent euthanization 48 h after intervention by cervical dislocation. Each heart was removed, embedded in O.C.T.^TM^ Compound (Sakura Finetek, Alphen aan den Rijn, Nederlands) and snap-frozen in liquid nitrogen. For further examinations of the infarction area [13] and RT-PCR, the heart tissue was divided into four horizontal levels from the apex to the base and cut into slices.

### 2.12. Immunofluorescence Staining within Tissue Sections

For immunofluorescence staining slices of cryosectioned hearts were fixed with 4% paraformaldehyde (Sigma Aldrich) for 20 min at room temperature following treatment with M.O.M.^TM^ Mouse Ig Blocking Reagent and M.O.M.^TM^ protein concentrate according to the instructions of Vector^®^ M.O.M.^TM^ Immunodetection Kit (LINARIS GmbH, Mannheim, Germany). To identify human cells, monoclonal anti-human-nuclei (Merck Millipore, Darmstadt, Germany) primary antibody as first and anti-mouse Alexa-Fluor^®^ 594 as secondary antibody was used. Nuclei were stained with DAPI and the tissue was mounted using coverslip and Dako mounting medium (Dako, Santa Clara, CA, USA).

### 2.13. Gene Expression Analysis by RT² Profiler PCR Array

Interlayers of the cryosectioned hearts were used for RNA-isolation, which was performed with TRIzol^®^ reagent. Reverse transcription as well as PCR were performed using RT² Profiler PCR Array Mouse Angiogenesis^®^ kit (Qiagen, Germantown, MD, USA). According to the instructions of the manufacturer an RT^2^ SYBR Green ROX qPCR Mastermix (Qiagen) was used. The expression of the housekeeping genes *ActB*, *B2M*, *GAPDH*, *GUSB*, and *HSP90AB1* as well as the gene panel were analyzed in triplicates and normalized to the mean of all the housekeeping genes. No genomic DNA was found in the samples. Moreover, we observed no apparent inhibition by any impurities that affect the reverse transcription as well as the amplification of the gene-specific products.

### 2.14. Statistical Analysis

Statistical analysis was performed using SigmaStat (Version 3.5, Systat Software, San Jose, CA, USA) and IBM SPSS Statistics for Windows (Version 22.0., IBM Corp., Armonk, NY, USA). Comparisons of two experimental groups were performed using Mann–Whitney *U* test, when data were not normally distributed (Shapiro–Wilk’s test) otherwise Student *t*-test was utilized. *p* values ≤0.05 were considered as statistically significant.

## 3. Results

### 3.1. Immunomagnetic Separation of CD133^+^ and CD271^+^ Cells from BM

**Figure 1:**
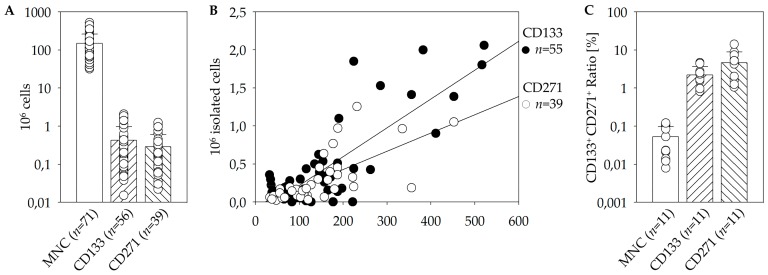
Quality of the immunomagnetic cell separation of CD271^+^ and CD133^+^ cells from fresh bone marrow (BM). Mean cell numbers of mononuclear cells (MNC), CD133^+^, and CD271^+^ cells achieved by magnet activated cell sorting (MACS) direct cell isolation (**A**). Linear dependence of CD133^+^ as well as CD271^+^ cell numbers on the initial amount of MNC (linear regression plots: R_CD133_ = 0.821; R_CD271_ = 0.689; **B**). The percentage of the CD133/CD271 double-positive cells determined within the different populations MNC, CD133^+^, as well as CD271^+^ cells using flow cytometry (**C**).

In order to optimize the procedure of cell isolation it had to be determined which impact the dual isolation strategy has on the cell product quality. Therefore, the CD133^+^ population was isolated from the CD271^–^ fraction as well as vice versa. Thus, the quality of the cell populations resulting from the isolation was compared with each other. The average volume achieved by BM aspiration was approximately 53.8 mL. By the use of the manual immunomagnetic cell separation 150 × 10^6^ MNC were isolated on average. From these a mean of 0.42 × 10^6^ viable CD133^+^ and 0.29 × 10^6^ viable CD271^+^ cells were separated (Figure 1A). Furthermore, analysis revealed that the amount of CD133^+^ as well as CD271^+^ cells showed a linear dependence on the initial amount of MNC that has been used for isolation (Figure 1B).

### 3.2. Flow Cytometric Characterization of Stem Cell Populations, Quality, and Storage

Furthermore, we have examined whether CD133^+^ and CD271^+^ cells form separate populations within BM or if the surface proteins are also expressed simultaneously by an overlap population, which is an important aspect in order to evaluate the feasibility of a combined therapeutic approach. Taking into account that the majority of CD133^+^ cells express CD34^+^, MACS-purified CD133^+^ cells were co-stained with CD34 antibody [14,15]. Moreover, the CD271^+^ population was co-stained for CD45 for further distinguishing of the MSC subpopulation lacking the CD45 surface marker [16]. As demonstrated below, 0.04 ± 0.01% of BM-MNC co-expressed CD133 and CD271. This subpopulation represents 2.21 ± 0.43% of total CD133^+^ stem cells and 4.65 ± 1.68% of total CD271^+^ stem cells (Figure 1C). As another aspect of cell isolation, the impact of the dual isolation strategy was investigated. Therefore, the CD133^+^ population was isolated from the CD271^−^ fraction and vice versa. The respective comparisons of the qualities between the obtained subpopulations were conducted. We found that 1.14 ± 0.50% of the isolated CD133^+^ population were positive for CD271, and 3.00 ± 0.99% of the isolated CD271^+^ cells were also positive for CD133. At this point, no indication that the order of isolation has a negative or significant influence on the quality or cell number was detected.

Additionally, the impact of the storage on the cell quality versus the quality of the fresh samples was examined. In the clinical scenario, the BM samples reach the laboratory depending on the time the heart surgery is performed, which cannot always be predicted precisely. Considering the fact that one single MACS-isolation takes around five hours, the possibility of sample storage would make experimental work much easier and effective. Furthermore, it is unavoidable that the cells undergo storage for at least several hours due to processing and transportation. This applies for storage of BM intended for mono-cell type isolation as well as for negative fractions assigned for dual isolations. We could find that overnight storage of the initial material at 4 °C had no significant negative influence on numbers or quality of the target cells resulting from the isolation (Table 1).

### 3.3. In Vitro Network Formation

**Figure 2:**
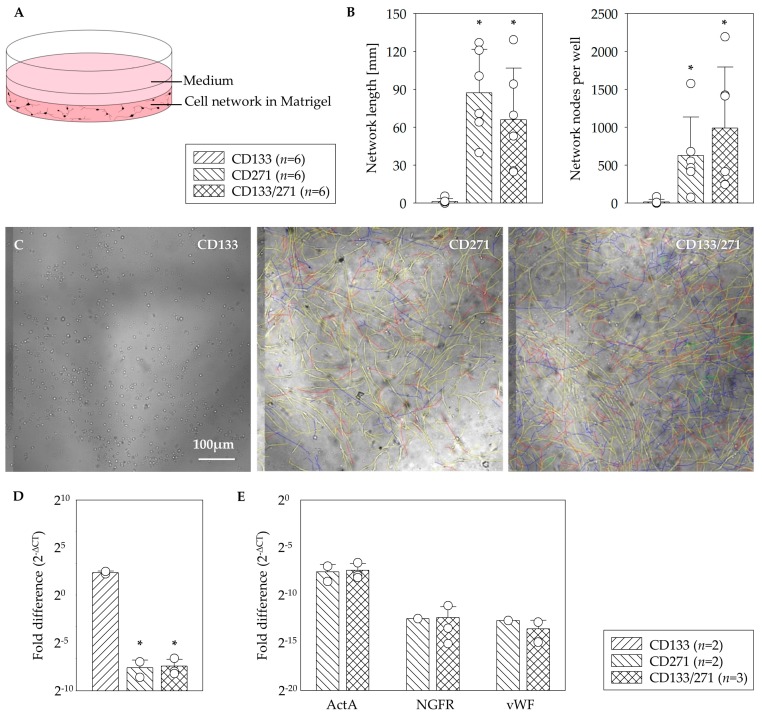
In vitro angiogenesis assay. Schematic illustration of the network formation assay within the Matrigel for testing of the angiogenesis potential (**A**). In contrast to CD133^+^ cells, a significant formation of network length and highest number of nodal points in the network of CD271^+^ cells as well as CD133^+^/CD271^+^ cell co-culture were observed (**B**). Representative phase contrast z-stack images of the mono- and co-culture assays (**C**). CD133^+^ cell culture in Matrigel led to a 10-fold higher level of *ActA* gene in comparison to CD133^+^/271^+^ cell co-culture (**D**). No differences were observed between CD271^+^ and CD133^+^/271^+^ cell culture assays for *ActA*, *NGFR,* and *vWF* gene expression (**E**). Mean ± SD; * *p* ≤ 0.015 vs. CD133, Mann–Whitney *U* test.

In order to investigate to what extend the stem cells build networks of endothelial cells and thus to evaluate their angiogenic capabilities, the isolated fractions of CD133^+^ and CD271^+^ cells were cultivated in Matrigel and EGM-2. Matrigel was used to create a complex extracellular environment with structural proteins like laminin, entactin, and collagen which resembles natural conditions. For the purpose of stimulating the cell growth and endothelial differentiation EGM-2 was used as an endothelial growth medium (Figure 2A). Each patient’s CD133^+^ and CD271^+^ cells were seeded separately in order to compare their tendency to build network structures. Additionally, one assay consisting of both cell types in equal amounts was created with the aim of investigating whether they influence each other and alter the network formation. All assays contained cells of one patient only. The angiogenesis assays were analyzed according to the criteria of network length and count of nodal points using two-dimensional transversal slice images of the three-dimensional assay (Figure 2B) as well as in regard with levels of the gene expression for *ActA*, *NGFR,* and *vWF*. This allowed representative analysis of the structures (Figure 2C). The CD271^+^ cells formed well-defined, 3D networks within Matrigel matrix, whereas CD133^+^ cells only have formed minimal networks in three out of six experiments at all. The assays consisting of CD133^+^/CD271^+^ cells in co-culture also showed well-defined, 3D networks. CD271^+^ cells generated networks with mean lengths of 81.20 ± 86.02 mm, combined CD133^+^/CD271^+^ cell culture assays formed networks with 60.35 ± 44.81 mm in length, and CD133^+^ cells only formed 1.5 ± 4.74 mm of such network structure. Thus, in terms of network length CD271^+^ alone and assays of the co-culture consisting of CD133^+^/CD271^+^ cells were both significant compared to CD133^+^ cells alone. Moreover, networks formed by CD133^+^/CD271^+^ cells in co-culture contained the highest number of nodal points (893.2 ± 838.4), which revealed as significant when compared to CD133^+^ cells alone (18.1 ± 75.4). The amount of nodal points built in CD271^+^ cell networks regarded also as significant in comparison with those formed in CD133^+^ cell assay (639.9 ± 901 vs. 18.1 ± 75.4, respectively). A quantitative mRNA-expression analysis demonstrated a 10-fold higher level of the Actin assembly-inducing protein ActA in CD133^+^ cell monoculture compared to CD271^+^ cell influenced assays (Figure 2D). No differences were observed between CD271^+^ and CD133^+^/CD271^+^ cell assays for *ActA*, the *NGFR* as well as the angiogenic marker *vWF* genes expression (Figure 2E).

### 3.4. Identification of Stem Cell Phenotype

**Figure 3:**
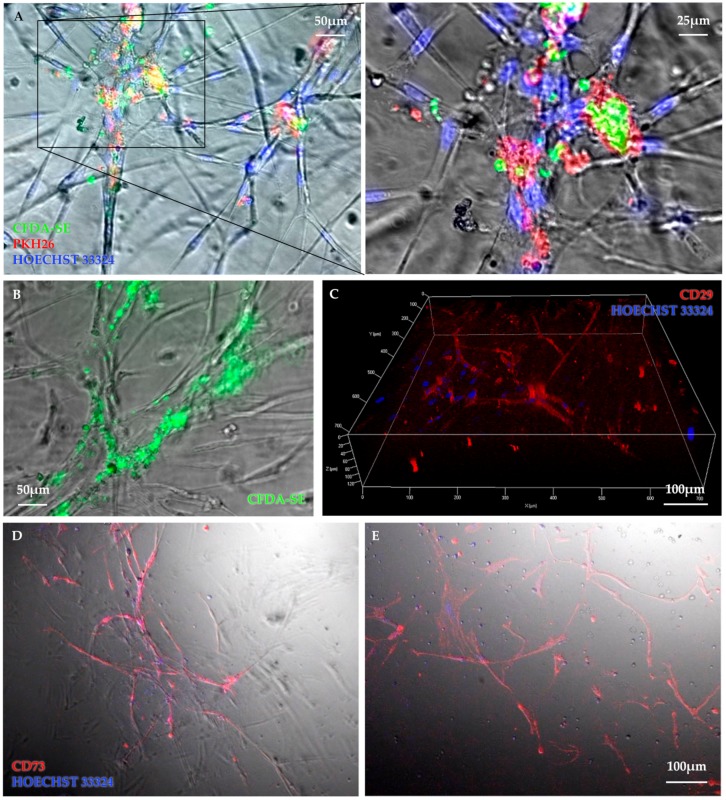
Representative images illustrate cellular interactions within the network structure. Colocalization of CFDA-SE labelled CD133^+^ cells (green) and PKH26-labelled CD271^+^ cells (red) at nodal points of the CD133^+^/CD271^+^ cell co-culture angiogenesis assay but with very rare or no connections of CD133^+^ cell culture were observed (**A**). Although CD133^+^ cell cultures built minimal networks and true CFDA-SE fluorescent signal originate inside the network structures (**B**). 3D-image of a vital mesenchymal cell network was taken 2 weeks after seeding (**C**). CD271^+^ cell (**D**) as well as CD133^+^/CD271^+^ cell co-culture (**E**) angiogenesis assays were stained with CD73-PE antibody (red) and counterstained for nuclei with Hoechst dye (blue).

In order to further investigate the cell networks accomplished in Matrigel, respective immunohistochemical analysis was carried out. Therefor cells were labelled with different fluorescent dyes, allowing the structures to become more perceptible and differentiable. In this regard, the assays containing both cell types in co-culture were of particular interest. By using different dyes for the stem cell subpopulations the staining should reveal whether both cell types take part in building the network structures and to which extent. The CD133^+^ and CD271^+^ cells have been labelled with CFDA-SE and PKH 26, respectively. Both fluorescent dyes could be detected within the network structures. Figure 3A shows a colocalization of CFDA labelled CD133^+^ cells and PKH26 labelled CD271^+^ cells at nodal points of the CD133^+^/CD271^+^ cell co-culture angiogenesis assay but no connections were observed within the CD133^+^ mono cell assay. Of note, the true CFDA-SE fluorescent signals come from inside of the cellular extensions (Figure 3B) although CD133^+^ cell monocultures built minimal networks. In turn, it demonstrates that CD133^+^ cells form networks when cultured with CD271^+^ cells. To obtain information on the stem cell differentiation degree and phenotypic fate within the network, differences in the extent and distributions of the subpopulations in those assays have been tested. At this, the cells were positive for CD29 (Figure 3C). The same applies to the staining of CD271^+^ cell culture with CD73 (Figure 3D). Conversely, the staining with CD271 antibody and its isotype control were both negative. Based on these results, the CD133^+^/CD271^+^ cell co-culture network showed CD73 expression (Figure 3E). Additionally, no signals for CD73 were detectable in assays lacking network formation.

### 3.5. In Vivo Angiogenic Benefit

**Figure 4:**
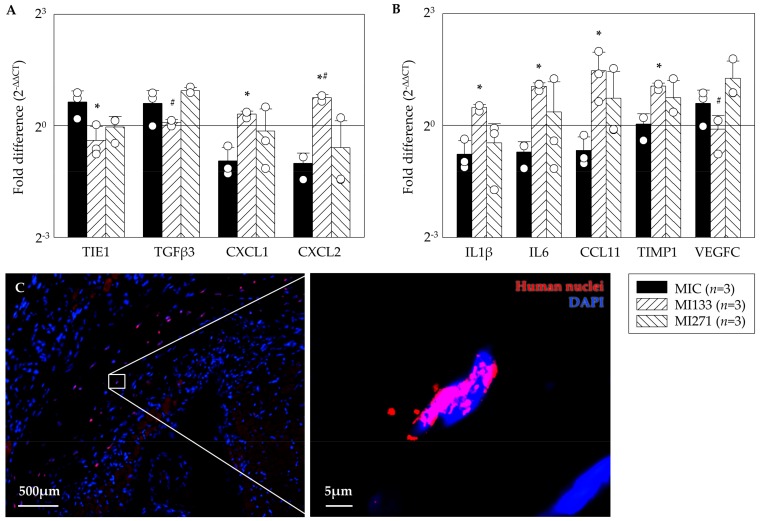
Angiogenic benefit 48 h after myocardial infarction (MI). Data show a significant lower expression of fibrotic markers as well as significant improvement of pro angiogenic factors after CD133^+^ cell treatment compared to MIC as well as MI271 group (**A**). Lower expression of inflammatory cytokines after CD271^+^ stem cell treatment in contrast to significantly increase after CD133^+^ stem cell treatment as well as significantly improvement of the pro angiogenic factor VEGFC (vascular endothelial growth factor) after CD271^+^ cell treatment. (**B**). Mean ± SD normalized to SHAM operation; * *p* ≤ 0.05 vs. MIC; # *p* ≤ 0.05 vs. MI271 (*t*-test). Representative images of CD271^+^ cell derivatives illustrate the engrafted human stem cells 48 h post transplantation (**C**).

Currently, animal experiments are necessary to evaluate the effects of stem cell therapies on the infarcted hearts. The complex pathophysiological processes have to be taken into account, as the intact circulation, vegetative nervous system, and cellular interactions of the entire organism are essential to guarantee reliable scientific results. By the use of a small animal model, applied stem cells were investigated regarding their capability to differentiate as well as which gene expression patterns were associated. For quantitative assessment of the angiogenic benefit we performed Real-Time-PCR assays using the RT² Profiler PCR Array Mouse Angiogenesis^®^ kit. The ΔΔC_T_ method was used to calculate fold-differences in target gens compared to the SHAM operation. Following CD133^+^ cell treatment (MI133) we observed a significant lower expression of the inflammatory cytokine TIE1 (Tyrosine kinase with immunoglobulin-like and EGF-like domains) and fibrotic marker TGFβ3 and a significant improvement of the pro-angiogenic factors such as CXCL1 and CXCL2 compared to MIC as well as MI271 group (Figure 4A). After CD271^+^ cell treatment we found a lower expression of the inflammatory cytokines IL1β, IL6, CCL11, and TIMP1 in contrast to a significant increase after CD133^+^ stem cell treatment versus MIC and the angiogenic factor VEGFC (vascular endothelial growth factor) was significantly improved compared to MI133 (Figure 4B). We were able to find the transplanted human cells 48 h after transplantation at the border of the infarct (Figure 4C).

## 4. Discussion

Our present study predominantly relied on investigation of angiogenic capabilities of primed human CD133^+^ and CD271^+^ BM derived stem cells alone and in a co-culture in vitro as well as in mice using combined cell transplantation post MI. Recently, we already have demonstrated the beneficial therapy when a single administration of stem cells was applied [13].

First, we were able to achieve suitable isolation protocols and storage parameters. The results from the manual immuno-magnetic cell separation in combination with flow cytometric analysis revealed relatively few CD133^+^ and CD271^+^ cells within BM. Moreover, a linear dependence between target stem cells and the initial amount of MNC has been found (Figure 1B). However, the initial cell numbers and percentages differed eminently within the patient’s cohort [17,18].

Whether CD133 and CD271 are separate subsets or co-expression exists, is an important aspect in order to evaluate the feasibility of a combined therapeutic approach. In conclusion, CD133^+^ CD271^+^ double-positive cells are represented at a higher proportion within the CD133^+^ as well as the CD271^+^ cells than within the total MNC fraction (Figure 1C). Nevertheless, only a small percentage of isolated cells express both surface markers. During magnetic cell separation no selective enrichment or depletion of double or single positive cells took place. Therefore, the separation of the two populations is considered to be sufficient for this study and the investigation of their separate and combined effects.

In clinical setting, autologous stem cells may be transplanted 24 to 30 h after BM aspiration [19]. A recent study by our laboratory closely investigated the impact of short-term storage on human CD133^+^ cells. At this, we used standardized non-freezing storage conditions even for up to 72 h. We have shown that cell number as well as metabolic activity decreased after 30 h, although no significant alterations were observed in cell viability at this point over the time. In line with that, also present results do not indicate a negative influence of storage on cell numbers or quality (Table 1). This applies to storage of BM as well as for negative fractions intended for dual isolation.

In the present study, we have characterized the phenotype of both stem cells types. The flow cytometric strategy relied on our previous work [13,15]. Cultivated MSC are often characterized by the expression of several surface marker including CD73, CD105, and CD44 as well as the absence of others, including CD45 and CD34 [2,13]. In our analysis the expression of CD73 and CD105 was significantly increased on CD271^+^ cells compared to remaining BM subtypes. We have already detected a favorable survival pattern, improved healing performance, and a more robust preservation of cardiac function for CD105^+^ cells in infarcted hearts [20]. Therefore, the increased expression of CD105 on the CD271^+^ cell population can be expected to have a positive influence on the angiogenic potential. Whereas most of cultivated MSC do not express CD45, the largest proportion of freshly isolated CD271^+^ cells expressed CD45^dim^ - a surface marker that has been correlated to mesenchymal colony formation by Cuthbert and coworkers [16]. The percentage of CD45^-^ cells was small and showed considerable fluctuations between patients. In conclusion, the most frequent population in purified CD271^+^ cells presumably consists of mesenchymal progenitors [13]. Of note, as our recent flow cytometric observation revealed, the same CD271^+^ MSC used for the present study have undergone phenotypic alterations toward a pericytic phenotype [13]. This is in agreement with the observation by Kutcher and coworkers and Bellagamba and associates [21,22].

Proliferative capacity of MSC is associated with their clonogenic properties. In accordance with Quirici and coworkers [10], colony-formation was only detected in MNC and CD271^+^ fraction, but neither in CD271^–^ nor in CD133^+^ population [13]. The ability to proliferate is a major contributor to the impacts and effectiveness of stem cells in clinical applications. This applies to various functions such as differentiation into specialized mature cells as well as self-renewal or angiogenic processes. Although CD133^+^ HSC do not show colony formation in CFU-F assays, they have been demonstrated to proliferate and generate offspring with endothelial characteristics in CFU-EC assays [19]. CFU-EC acquire the functional phenotype of EC and also show typical gene expression including vWF [19] and angiogenic factors such as FGF3, PDGFB, and others [23]. Furthermore, we detected a significant augmentation of EC-colonies after hypoxic preconditioning of CD133^+^ cells and thus assumed that hypoxia may lead to the differentiation of CD133^+^ cells towards endothelial lineage [23]. Our results indicate that in vitro network formation relies on CD271^+^ cells. Since CD133^+^ cells alone rarely build network structures at all, it is remarkable that they will nevertheless contribute to the network, if they are cultivated in combination with CD271^+^ cells (Figure 2). The fluorescence images provided evidence for this contribution as both fluorescent signals were detected from within the networks (Figure 3). Noteworthy, we previously observed similar network formation also in co-culture when primed cardiac CD45^-^ CD44^+^ DDR2^+^ MSC from MI-induced rats were setup together with endothelial cells [24]. Immunoflorescence staining indicated a strong expression of CD73 marker within the networks built by CD271^+^ as well as CD133^+^/CD271^+^ cells of the angiogenesis assays. CD73 is often referred to us the MSC phenotype [25,26]. CD133^+^ cell networks also expressed CD73 whereas the maker was not detected on CD133^+^ cells that did not form network structures. For this study the involvement of the marker in angiogenic processes is of particular importance. At this, CD73 does not only seem to be associated with cells that are involved in angiogenesis, but appears to play a crucial role in the process itself. A study by Wang and associates found that capillary-like structures were formed more in CD73^+/+^ pulmonary microvascular endothelial cells (PMEC) than in CD73^−/−^ PMEC in vitro. In accordance with that they also observed that the extent of tumor angiogenesis as well as the tumor size was greater in CD73^+/+^ mice compared to CD73^−/−^ mice in vivo. Furthermore, CD73 expression decreased the adhesion of EC to collagen IV and promoted migration. Thus, they concluded that CD73 contributes to EC forming new vessels especially in cancer conditions [27]. A study by Allard and associates demonstrated that both tumor and host-derived CD73 are involved in these processes. In fact, tumor-derived CD73 enhances the production of vascular endothelial growth factor (VEGF) by tumor cells whereas host-derived CD73 is required for in vivo angiogenic responses. Furthermore, they also confirmed that EC require CD73 expression for tube formation and migration [28]. Consequently, measures against CD73 have been shown to impair angiogenesis in tumors [28,29]. However, Böring and associates stated that CD73 deficiency has no effect on angiogenesis in their mice model of hind limb ischemia [30]. Thus, it is not certain if the angiogenic effect applies on tumor tissue only and might be different in non-tumor environment. The increased expression of CD73 especially on CD271^+^ cells may have positive impact on the angiogenic potential of the stem cell treatment. We were able to show that CD271 network structures express ActA and thus a marker for muscular differentiation. This expression was 10 fold higher in assays consisting of CD133^+^ cell cultures (Figure 2D). To simulate network formation in vitro, we used Matrigel as a 3D framework. Interestingly the CD133^+^ cell culture in Matrigel led to a 10-fold higher level of *ActA* (*α-SMA*) gene in comparison to the CD133^+^/271^+^ cell co-culture. However, Lu and associates observed that following culture in endothelial cell-promoting environment CD133^+^ cells did not produce endothelial-like cells that expressed α-SMA, but only HUVEC and CD34^+^ progenitor cells [31]. As we have already shown recently, flow cytometric analysis of the CD133^+^ cell product isolated from sternal BM (similar patient’s disease baseline as in the present study) was characterized by the co-expression of approx. 80% of CD34 marker [15]. Therefore, detected *ActA* mRNA levels in our CD133^+^ cell angiogenesis assay seem to be reasonable. Interestingly, CD133^+^ αSMA^+^ phenotype was found only in association with blood vessels of cancer-associated fibroblasts [32], confirming the linkage of α-SMA expression to endothelial-like origin and cell activation. Furthermore, the possible reason of less or comparable *ActaA* mRNA levels with a CD271^+^ cell culture for the CD133^+^/CD271^+^ cell co-culture might be explained by the phenotypical alterations of CD133^+^ hematopoietic stem cells when applied together with MSC and/or due to the maintained undifferentiated state of MSC in co-culture model [33,34]. Hence, these in vitro co-culture conditions may have a reducing effect on the *α-SMA* mRNA levels in total.

Moreover, we examined mRNA expression profiles using the infarcted area of heart cross sections. In line with recent observations of the proliferative capacities and induction of regenerative pathways, demonstrated by our group [24], the present study focused on testing of possible angiogenic potential in the early phase post MI. On the one hand in a murine model, the switch between the early inflammatory and the proliferative phase eventuate 2-4 days after MI [35]. At the same time, neovascularization of surrounding, viable myocardium in the infarct border zone is also crucial during this process of tissue remodeling, although angiogenesis occurs in the granulation tissue that will ultimately form the infarct scar [36]. On the other hand, a complete engraftment and no significant differences in the retention of both cell subsets were evident in the hearts 48 h following MI as shown in our own previous work [13]. Indeed, in a recent long term study using the identical human stem cell types as well as the identical mouse model we did observe functional improvement [13].

Recently, we could show the co-localization of CD133^+^ cell derivatives with functional blood vessels 3 weeks post transplantation. This direct impact of CD133^+^ stem cells on angiogenesis was not detected for CD271^+^ cells, suggesting different cellular mechanisms for cardiac regeneration [13]. Coherently, indications of the present study 48 h after MI reveal positive effects of the stem cell therapy regarding angiogenesis. The expression of the pro-angiogenic cytokines Cxcl1 and Cxcl2 has been significantly increased in the CD133^+^ cell treated animal group, whereas the MI271 group showed less expression (Figure 4A). However, the same applies to the expression of IL1B and IL6 as well as Timp1 [37] (Figure 4B). Al-Amran and co-workers demonstrated that down-regulation of Cxcl2 results in decrement in myocardial neutrophil infiltration, which is associated with less reactive oxygen species and reactive nitrogen species formation after global ischemia reperfusion apoptosis [38]. Therefore, we assume, that CD133^+^ cell therapy may have a valuable impact on a moderate course of the neutrophil-mediated tissue injury [39], although, we only could observe a significant lower *TGFβ3* mRNA level of this fibrotic marker in MI133 therapy group when compared with MI271 [40]. Our long term follow up experiments revealed unanticipated data. Subsequently, both cell therapy groups led to a decreased collagen deposition at the infarction border as well as improved vessel infiltration of scar tissue compared to the MIC group [13,40,41,42]. Again, these data show possible different cellular mechanisms for angiogenic effects mediated via CD133^+^ vs. CD271^+^. Furthermore, many existing in vitro studies confirmed the involvement of TGFβ in the activation of cardiac fibroblasts. Nevertheless, the in vivo functioning has not completely been explained because of the complexity and the context-dependent signaling cascades [40]. Interestingly, mRNA levels for pro-inflammatory interleukins (see above) within MI133 therapy group were significantly enhanced, while TGFβ3 levels were rather reduced. Since IL-6 bears pleiotropic properties, a possible switch from pro- into anti-inflammatory or even immunomodulatory signaling cascades, would be in favor for a respective therapy. Importantly, it has been shown, that both IL-6 with TGFβ play a crucial role in the shift between inhibitory Tregs and pro-inflammatory Th17 cells and this is associated with their ratio at all [43].

Finally, we have observed significantly increased mRNA levels of the pro angiogenic factor VEGFC after CD271^+^ stem cell treatment compared to MI133 group (Figure 4B). It is known that MSC promotes angiogenesis by releasing VEGF [44,45,46]. Accordingly, Liu and associates observed enhanced cardiac function, increased perfusion and angiogenesis in vivo using MSC as a VEGF delivery system [47]. Collectively, analysis of hearts of the MI271 therapy in the early ischemic phase revealed rather immunomodulatory fate of CD271^+^ stem cells.

## 5. Limitations

It must be stated that the present study and the generated data rely on a relatively small sample size. Thereby, sternal bone marrow for the present study was obtained from patients with CABG surgery. In general, we were able to perform standard quality control experiments at a high sample size (Section 3.1 and Section 3.2). However, downstream use of these samples served to address various related projects, resulting in a limited sample size for the respective specific experiments.

## Figures and Tables

**Table 1:** Influence of the storage (4 °C) and order of the isolation during the dual isolation of CD133^+^ or CD271^+^ cells on their cell numbers and quality. Single MACS isolations of CD133^+^ or CD271^+^ cells were utilized on fresh (I) or stored (II) BM samples. For dual isolations, respective negative fractions (III or IV) were stored and respective target isolations (CD133^+^ or CD271^+^) were conducted after overnight storage.

	CD133^+^ Stem Cells						CD271^+^ Stem Cells					
	Count		Purity		Viability		Count		Purity		Viability	
	×10^6^	*n*	[%]	*n*	[%]	*n*	×10^6^	*n*	[%]	*n*	[%]	*n*
Single isolation												
I. Fresh BM	0.299	19	83.422	10	94.123	10	0.112	8	76.435	7	58.753	7
II. Storage BM	0.506	8	85.401	6	93.272	6	0.482	4	82.148	4	89.509	4
Dual isolation												
III. Storage CD133^–^ fraction	0.338	5	73.213	3	82.895	3	0.203	4	47.500	2	77.290	2
IV. Storage CD271^–^ fraction	0.137	7	88.292	3	96.182	3	0.170	7	88.430	6	89.824	6
